# Cesarean Section Scar Endometriosis: A Case Report From Sudan

**DOI:** 10.7759/cureus.90368

**Published:** 2025-08-18

**Authors:** Mohamed Elkhazein, Salma Ahmed, Mohamed B Ahmed, Mohammed A Ismail, Tafaol H Mokhtar, Ahazeej Gurashi, Hala Bashir

**Affiliations:** 1 Obstetrics and Gynaecology, Al-Butana University, Rufaa, SDN; 2 Obstetrics and Gynaecology, latifa Hospital, Dubai, ARE; 3 Surgery, Abu Hamad Teaching Hospital, Abu Hamad, SDN; 4 Obstetrics and Gynaecology, University of Medical Sciences & Technology, Khartoum, SDN; 5 Obstetrics and Gynaecology, Al-Hayat National Hospital, Khamis Mushait, SAU; 6 Obstetrics and Gynaecology, Al-Neelain University, Khartoum, SDN; 7 Obstetrics and Gynaecology, Latifa Hospital, Dubai, ARE

**Keywords:** cesarean, cesarean complications, cutaneous endometriosis, endometriosis, scar site endometriosis

## Abstract

Cesarean scar endometriosis is a rare form of endometriosis characterized by the presence of functional endometrial tissue within a surgical scar. Diagnosing this condition is challenging, as it is frequently misdiagnosed as granulomas, hernias, abscesses, hematomas, or neoplasms. The classic triad of symptoms includes a positive surgical history, cyclical pain, and a mass at the surgical scar. The presented case highlights the pivotal role of imaging modalities such as MRI, along with histopathological confirmation, in establishing a definitive diagnosis. Surgical excision with clear margins is the mainstay of treatment and remains superior to medical therapy in terms of long-term outcomes. Diagnosis relies on a high index of clinical suspicion. Clinical evaluation, along with ultrasound, computed tomography (CT), or magnetic resonance imaging (MRI), aids in diagnosis. Surgical intervention is the definitive treatment, providing specimens for histopathological confirmation. We report the case of a 27-year-old woman with a history of one cesarean delivery who presented with a progressively enlarging, painful mass over her lower abdominal scar. The pain was cyclical and associated with menstruation. MRI revealed a heterogeneous mass not reaching rectus sheath. Surgical excision of the lesion was performed, and histopathological examination confirmed the diagnosis of endometriosis. The patient recovered uneventfully, with complete resolution of symptoms and no recurrence at six-month follow-up. This case highlights the importance of considering scar endometriosis in women with a history of cesarean section who present with cyclical pain and a mass at the scar site. Early recognition and surgical excision are essential for symptom relief and to prevent recurrence. Increased clinical awareness is crucial to avoid misdiagnosis and unnecessary delays in treatment.

## Introduction

Endometriosis is a chronic, estrogen-dependent condition defined by the presence of functional endometrial-like cells and stroma outside the uterine cavity [1]. It affects approximately 10% of women of reproductive age and is most commonly found in pelvic organs such as the ovaries, fallopian tubes, uterosacral ligaments, and peritoneum [2]. However, in rare instances, endometrial tissue can implant in extrapelvic locations, including the gastrointestinal tract, urinary system, lungs, and surgical scars, resulting in atypical presentations that can pose diagnostic challenges [3,4].

Scar endometriosis is a rare form of extrapelvic endometriosis and typically occurs following obstetric or gynecologic surgeries that breach the uterine cavity. Cesarean section (C-section) is the most commonly associated procedure, and scar endometriosis has been reported in approximately 0.03-1.5% of women undergoing C-section [5,6]. The proposed mechanism involves the direct mechanical implantation of viable endometrial cells into the surgical wound at the time of uterine closure. These cells, under the influence of circulating estrogens, may proliferate and establish endometrial tissue within the scar [7]. Alternative theories include metaplasia of pluripotent mesenchymal cells and lymphovascular spread, although these are less supported in cases of isolated scar involvement [8].

Clinically, patients with scar endometriosis typically present with a firm, painful mass at or near the surgical incision site, often with cyclical exacerbation of symptoms during menstruation. However, some cases may lack a clear temporal pattern, further delaying diagnosis. Imaging modalities such as ultrasonography and magnetic resonance imaging (MRI) are useful in evaluating the lesion’s characteristics and extent, while histopathological examination remains essential for definitive diagnosis.

Despite its rarity, scar endometriosis should be considered in women presenting with abdominal wall masses and a history of C-section or other uterine surgery, particularly when symptoms exhibit a cyclical pattern.

The aim of this case report is to illustrate the clinical presentation, diagnostic pathway, and successful surgical management of a patient with cesarean scar endometriosis, while emphasizing the importance of early recognition and surgical awareness.

## Case presentation

**Figure 1:**
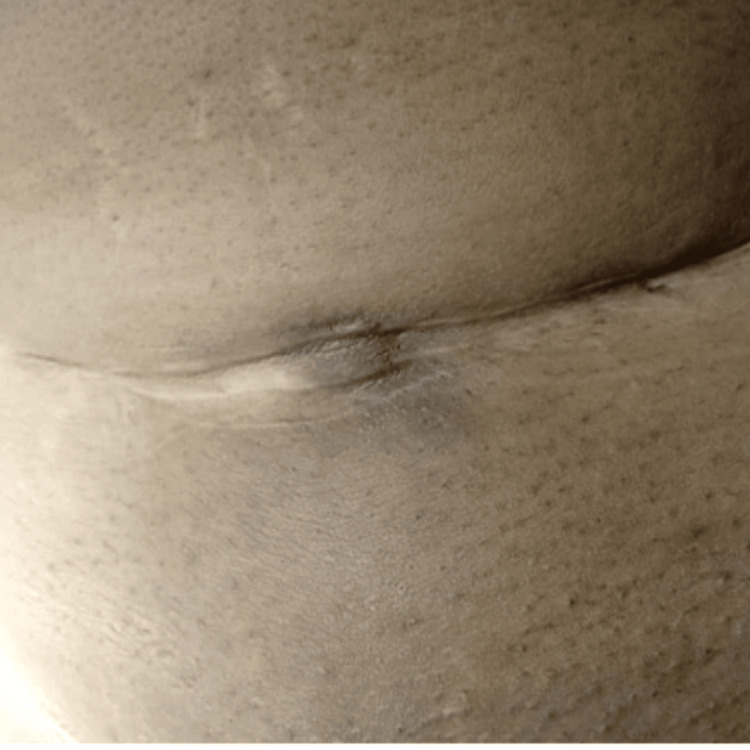
Scar endometriosis at the left lateral third of the caesarean section site

A 27-year-old multiparous woman with no significant medical history presented with intermittent lower abdominal pain. Then, she noticed a lump over her cesarean section scar after resuming her menstrual cycle, which changed color in relation to her menstrual cycle and became increasingly symptomatic (Figure 1). She had undergone an elective lower segment cesarean section three years prior for breech presentation, with an uneventful postoperative period. Her symptoms had gradually developed over eight months and intensified over the last two months, exhibiting a cyclical pattern.

She had no prior diagnosis of endometriosis and her menstrual history was unremarkable. Menstruation resumed one and a half years after her cesarean section. She had consulted multiple physicians, who initially diagnosed her condition as suture granulomas and treated her medically, but without improvement.

**Figure 2:**
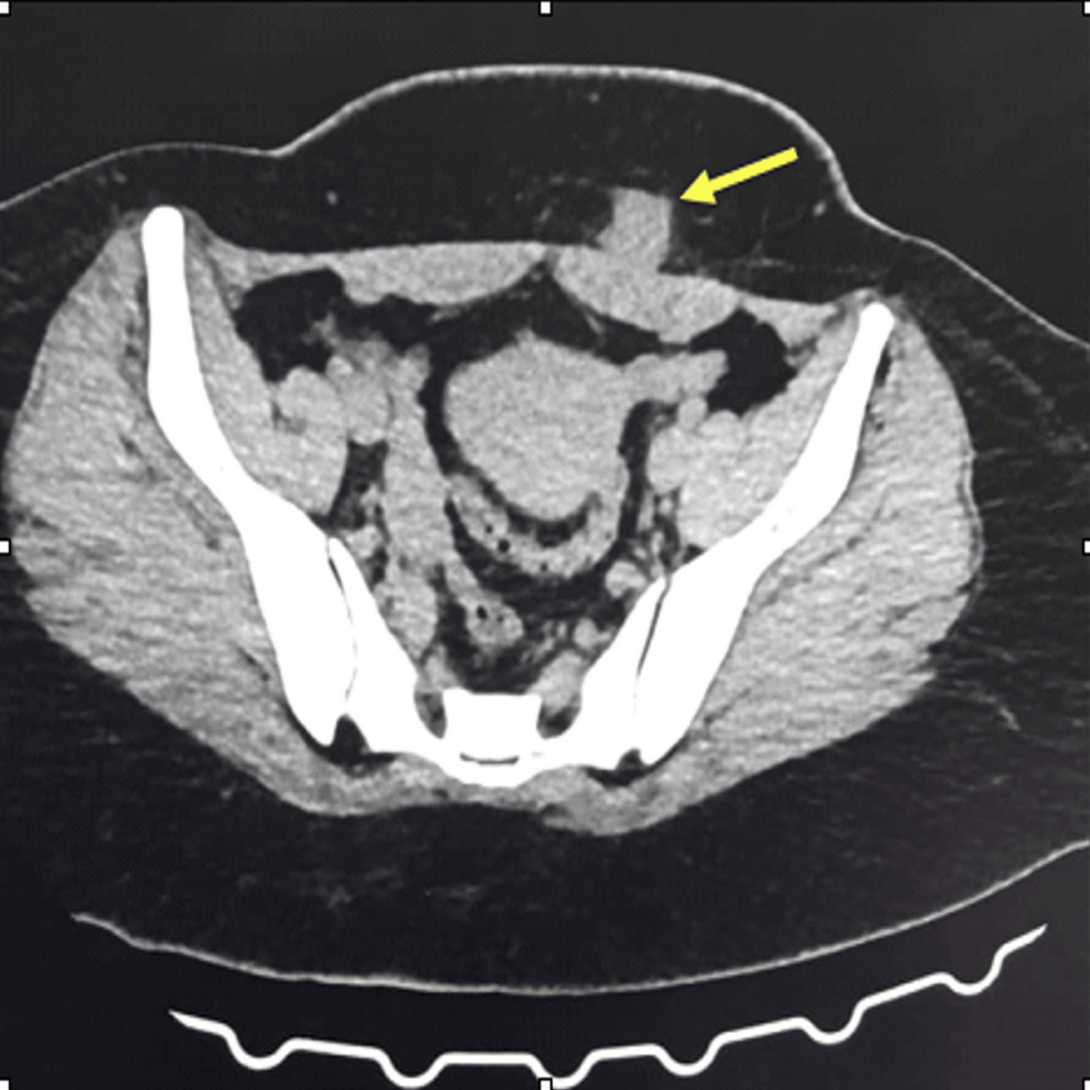
CT scan of the pelvis shows the lump at the lateral third of the scar site anterior to the left rectus muscle.

On clinical examination, a non-mobile, moderately pigmented, 2 x 2 cm nodule was observed at the left lateral third of the Pfannenstiel incision scar. It was tender on palpation. Ultrasonography revealed a bulky uterus and a well-defined hypoechoic lesion measuring approximately 1.9 x 1.8 cm in the subcutaneous plane beneath the surgical scar, with minimal vascularity-suggestive of scar endometriosis. Due to the unavailability of MRI, a contrast-enhanced CT scan of the abdomen and pelvis was performed (Figure 2). It identified an oval, hyperdense soft tissue mass measuring 2 x 2 cm in the subcutaneous fat of the left lateral third of the scar, anterior to the left rectus muscle but not infiltrating it. 

**Figure 3:**
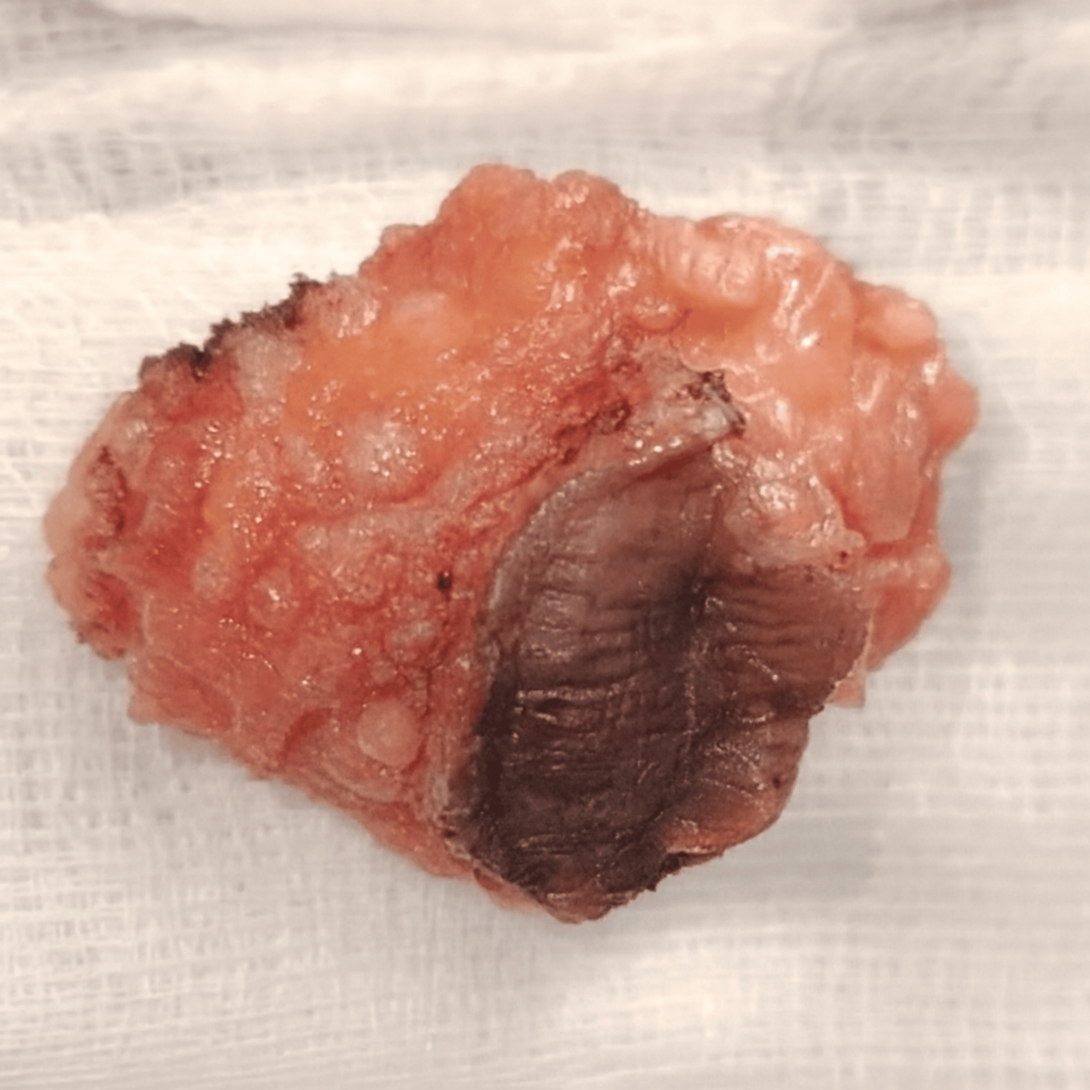
Tissues with the skin.

A clinical diagnosis of cesarean scar endometriosis was made based on the classic triad of symptoms. The patient opted for surgical excision, provided informed consent, and underwent surgery. The mass was located in the subcutaneous tissues and skin but did not invade the rectus sheath. The excised tissue was sent for histopathological analysis (Figure 3).

**Figure 4:**
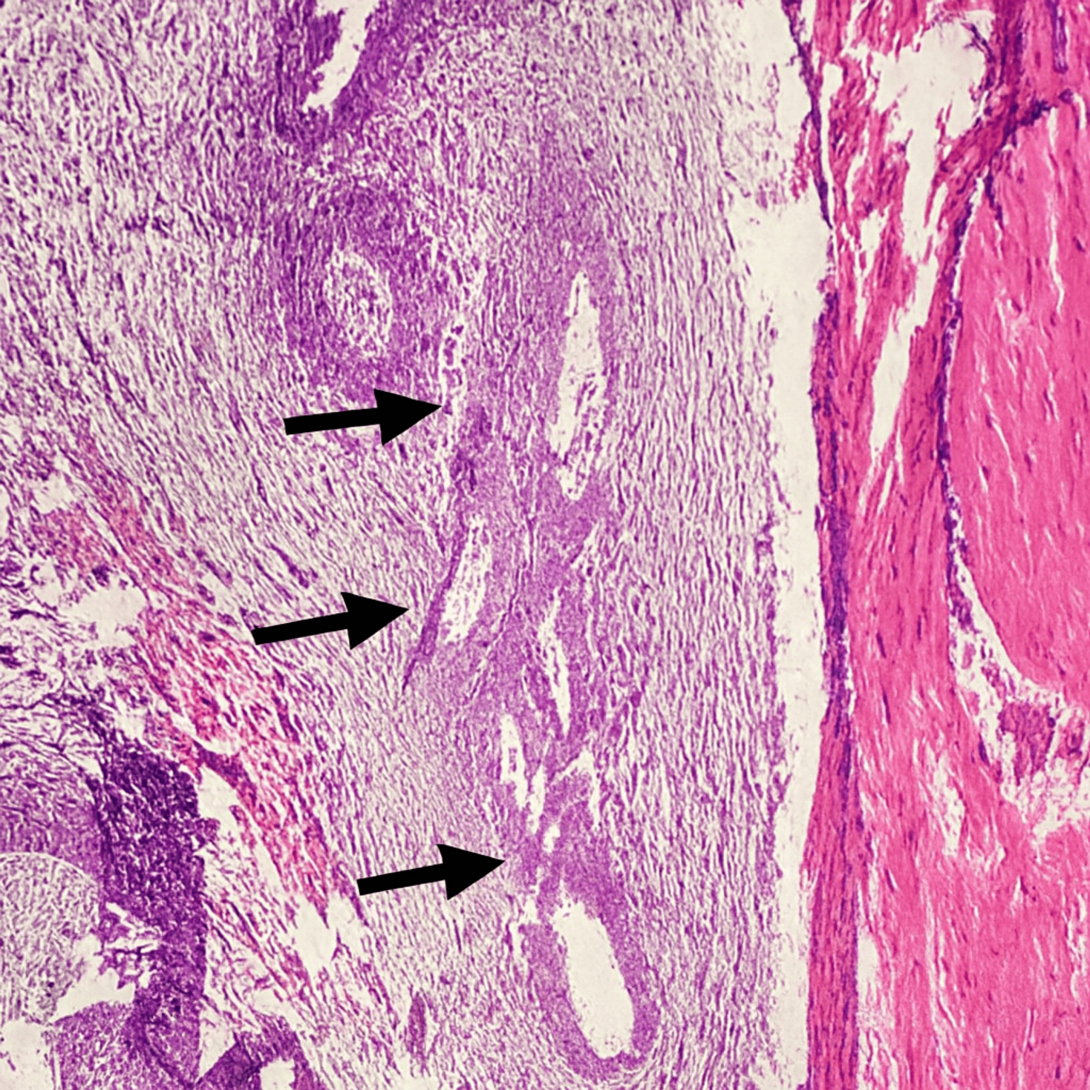
The excised tissues show a lesion composed of dilated endometrial glands and stroma embedded in fatty and fibrovascular stroma.

The patient was discharged the following day and followed up for three months, showing complete recovery. Histopathological examination confirmed the diagnosis of scar endometriosis, revealing a lesion composed of dilated endometrial glands and stroma embedded in fatty and fibrovascular tissue (Figure 4). No malignancy was detected. 

**Figure 5:**
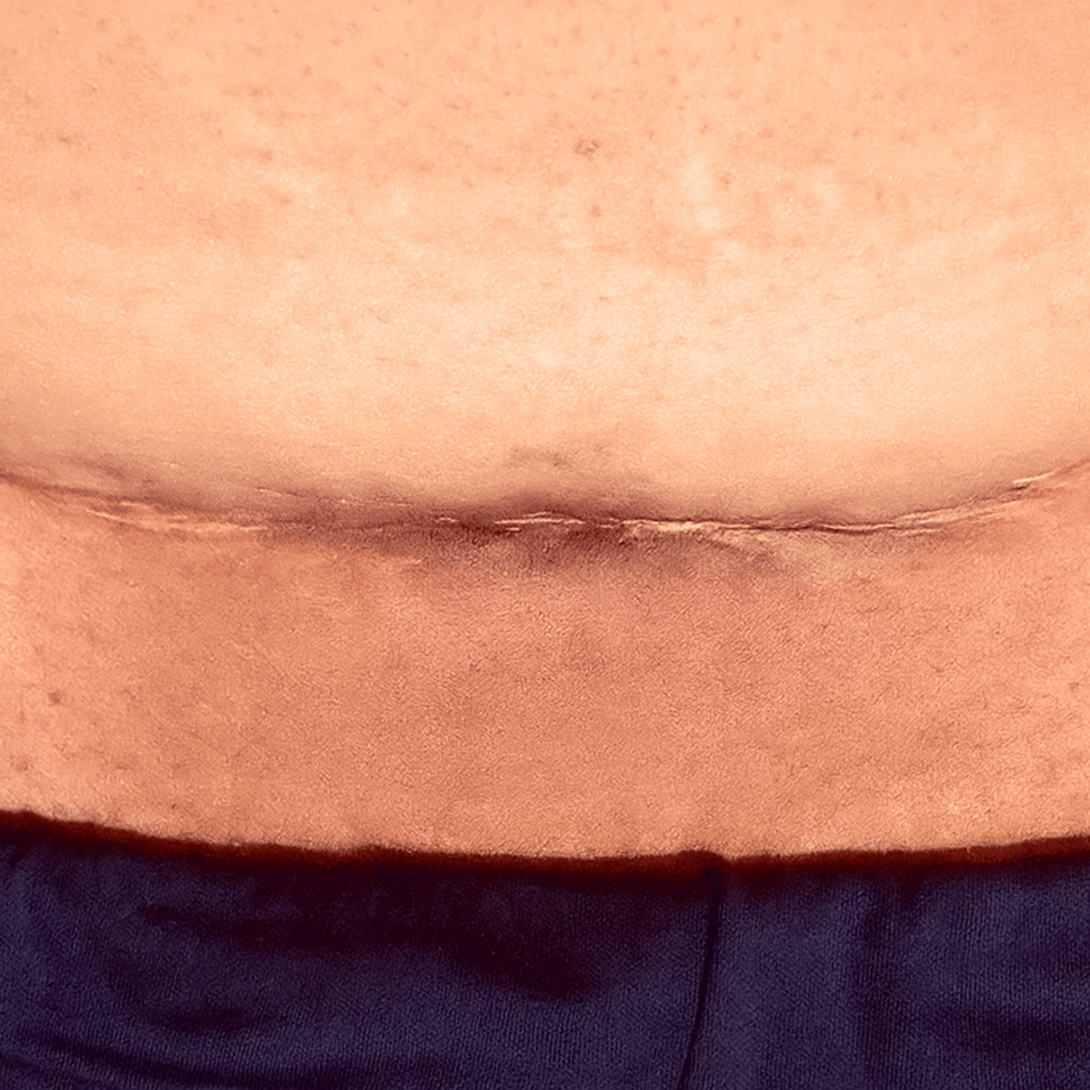
Scar healing on follow-up.

On follow-up, her surgical scar was well-aligned (Figure 5), and she had no further complaints.

## Discussion

Scar endometriosis is a rare but important differential diagnosis in reproductive-age women presenting with an abdominal wall mass, particularly following cesarean section. Our patient presented with a painful mass over her cesarean scar, with symptoms worsening during menstruation, consistent with the classical presentation described in the literature [1-4].

The pathogenesis is most widely explained by direct mechanical implantation of endometrial tissue into the surgical wound during uterine surgery, followed by proliferation under estrogenic stimulation [1,5,8]. Although other mechanisms, such as lymphatic or hematogenous spread and coelomic metaplasia, have been proposed [8], these remain less favored in scar endometriosis because of the strong anatomical and temporal association with surgical trauma.

Our patient’s diagnosis was delayed for several months due to the nonspecific nature of her symptoms, which is common in such cases [4,9]. While she exhibited cyclical pain, the mass was initially mistaken for a suture granuloma. Computed tomography (CT) was instrumental in identifying the lesion and delineating its size, margins, and relationship with surrounding structures, as MRI was not available at our center. The lesion was confined to the subcutaneous plane without involvement of the rectus sheath.

Fine-needle aspiration cytology (FNAC) has been advocated as a minimally invasive diagnostic option [6,10,11,12,13]. However, concerns about potential endometrial cell seeding along the needle tract remain [11,14]. Given these risks, and in line with other authors’ recommendations when clinical and imaging findings are strongly suggestive, we opted for definitive surgical excision without FNAC [4,10,11,12,13].

Complete surgical excision with clear margins remains the definitive treatment, offering the best chance for cure [1-4,9]. Mesh repair may be required for larger defects, although our patient did not require it. Histopathology confirmed the diagnosis of endometriosis, and she remains symptom-free at six months of follow-up. This outcome is consistent with reports in the literature, which describe low recurrence rates after wide excision [1,2,4,9].

Several recent reports mirror our findings. Kyejo et al. [9] and Paramythiotis et al. [10] documented latency periods of two to ten years between cesarean section and symptom onset, similar to our case. Triantafyllidou et al. [11] described rectus-sheath involvement, while Biegel et al. [12] reported a subcutaneous abdominal wall endometrioma after cesarean section, successfully treated with excision-paralleling our patient’s presentation and outcome. While MRI is often emphasized for lesion characterization and surgical planning [5,9,11,12], our case demonstrates that CT can provide adequate preoperative assessment when MRI is unavailable.

FNAC has been successfully used in some cases [6,10,11,12,13], but concerns about tract seeding persist [14]. For this reason, many authors, including ourselves, advocate for direct surgical excision when the diagnosis is strongly suspected and the lesion is operable. Wide local excision with clear margins is the standard approach, with mesh repair reserved for larger resections [1,4,9,11,12]. Recurrence is rare following complete excision [1,2,4,9]. Emerging alternatives, such as high-intensity focused ultrasound (HIFU), have shown promising symptom reduction in selected patients but remain experimental [15].

In summary, our case highlights the diagnostic challenges of scar endometriosis and underscores the importance of maintaining a high index of suspicion in women with prior cesarean delivery presenting with cyclical scar pain. Early diagnosis and appropriate surgical management can result in excellent outcomes and help avoid unnecessary interventions.

## Conclusions

Scar endometriosis, although rare, should be considered in the differential diagnosis of painful masses located in or near surgical scars, particularly in women of reproductive age with a history of cesarean section or other uterine surgeries. Delayed diagnosis is common due to nonspecific presentations, leading to prolonged patient discomfort and unnecessary investigations.

This case emphasizes the importance of clinical suspicion, especially when symptoms are cyclical and associated with prior uterine surgery. MRI was instrumental in our case for delineating the lesion's extent and guiding surgical planning, while histopathology confirmed the diagnosis. Wide local excision with clear margins remains the gold standard treatment, offering excellent outcomes and a low risk of recurrence. Hormonal therapies may offer temporary symptom relief but are not curative in isolated cases.

Preventive strategies during gynecological procedures, such as thorough irrigation of the surgical field and minimizing endometrial contamination, may help reduce the risk of implantation and subsequent scar formation.

This case highlights the need for heightened awareness among clinicians, particularly in primary care, surgery, and gynecology. Early recognition and timely referral for surgical management are crucial. A multidisciplinary approach involving gynecologists, radiologists, and surgeons ensures accurate diagnosis and effective treatment, ultimately improving patient outcomes and quality of life.
